# Role of SNAREs and Rabs in Myelin Regulation

**DOI:** 10.3390/ijms24119772

**Published:** 2023-06-05

**Authors:** Azzurra Margiotta

**Affiliations:** Department of Clinical Dentistry, Faculty of Medicine, University of Bergen, 5009 Bergen, Norway; azzurra.margiotta@uib.no

**Keywords:** SNAREs, rabs, neurotransmitter release, myelin

## Abstract

The myelin sheath is an insulating layer around the nerves of the brain and spinal cord which allows a fast and efficient nerve conduction. Myelin is made of protein and fatty substances and gives protection for the propagation of the electrical impulse. The myelin sheath is formed by oligodendrocytes in the central nervous system (CNS) and by Schwann cells in the peripheral nervous system (PNS). The myelin sheath presents a highly organized structure and expands both radially and longitudinally, but in a different way and with a different composition. Myelin alterations determine the onset of several neuropathies, as the electrical signal can be slowed or stopped. Soluble N-ethylmaleimide-sensitive factor attachment protein receptors (SNAREs) and ras (rat sarcoma)-associated binding proteins (rabs) have been proved to contribute to several aspects regarding the formation of myelin or dysmyelination. Here, I will describe the role of these proteins in regulating membrane trafficking and nerve conduction, myelin biogenesis and maintenance.

## 1. Introduction

The brain is an important organ which is fundamental to the regulation of many biological and physiological functions. It is divided into several regions which may encounter changes during development, maturation, normal aging, neurological disorders, and trauma. Together with the spinal cord, it forms the central nervous system. It is made of neurons, which store information and send impulses, and glial cells, astrocytes and oligodendrocytes, which support, insulate, and protect neurons. Neurons are very sensitive cells, with high demand for oxygen, glucose and other molecules. In order to work properly, neuron activity is based on the production of action potentials and the formation of ion gradients. Neurons use electrical impulses and chemical signals to send information to different areas of the brain and between the brain and the rest of the nervous system. Glial cells normally outnumber the neurons, and in the mature central nervous system (CNS) they consist of astrocytes, oligodendrocytes and microglial cells. In the peripheral nervous system (PNS) they are named Schwann cells, enteric glial cells and satellites cells [1]. Interestingly, it has been proved that astrocytes, microglia and oligodendrocytes contribute to modulating the neuronal synaptic structure and function. In fact, astrocytes have a role in neuronal activity by exhibiting excitability, due to the changes in intracellular calcium levels. They regulate neurotransmitter release, the extracellular environment and contribute to synapse elimination via phagocytosis or trogocytosis (synapse engulfment). Microglia can affect synapses by eliminating redundant neuronal dendritic spines and secreting microvesicles and specific molecules, such as cytokines and transmitters, while oligodendrocyte lineage cells can form functional synaptic contacts and regulate synapses in an unidirectional or bidirectional manner by releasing growth factors and neurotransmitters [2,3].

The transmission of the signal is dependent on the myelin sheath, which determines the speed of the information, and affects the capacitance/resistance and current flow. Myelin consists of multiple layers of specialized cell membrane that surround the axons larger than 1 µm in diameter. The myelin sheath covers the axons with the exclusion of the nodes of Ranvier (short regions of ~1 µm and <1% of the territory of the myelinated axon), which are small gaps between two adjacent myelin sheaths, small axons (<0.2–0.8 µm diameter), some large axons in the white matter, most of the axons in the grey matter [4,5], C-fibers in the PNS [6] and synapses [7].

Each myelin section constitutes an internode, which is normally 0.2–1 mm long. In the PNS, the length of the myelin sheath along the axon is around 1 mm. In the CNS, and in particular in anterior medullary velum of the adult rat, the internodal length is 50–750 µm [8]. However, myelin sheaths are vital for the formation, health, and function of the CNS, and its length is quite variable and fundamental to the axonal conduction velocity and the functional plasticity of neural circuits [9].

Small-diameter axons have a high number of internodes (as many as 80) ranging from 20 to 200 μm in length, while the oligodendrocytes that myelinate larger diameter axons have fewer processes but longer internodes and thicker myelin sheaths. The surface area of myelin of one oligodendrocyte might be 20 × 10^5^ µm [10].

The membranes of the myelin sheath are opposed and form the intraperiod line (IPL), a thin space that separates the membranes and which is contiguous with the extracellular space. On the other hand, the major dense line (MDL) is a 3 nm compartment between two cytoplasmic leaflets of stacked myelin membranes, where myelin basic protein (MBP) is abundant. 

The myelin sheath avoids the loss of signal and it is slightly different in the CNS compared to the one in the PNS. Despite being similar in structure, specific cell types (oligodendrocytes and Schwann cells) are responsible for the formation of myelin, in the CNS and PNS, respectively. Oligodendrocytes are responsible for the formation of up to 80 different layers of myelin membrane around the axons. The aim of the synthesis and maturation of these sheaths is a fast and efficient nerve conduction, while the maintenance and the remodeling of myelin are linked to the interaction between oligodendrocytes and glial and neuronal cells and its role in providing metabolic support to neurons, ion and water homeostasis, and adapting to activity-dependent neuronal signals [10].

Interestingly, it has been demonstrated that gap junctions and cytoplasmic diffusion pathways within the myelin are linked to the myelin sheath functions. The main types of voltage-gated channels are K^+^ channels, Cl^−^ channels, Na^+^ channels, Ca^2+^ channels and P2X ligand-gated ion channels. These ion channels confer with the myelin sheath specific electrophysiological properties. Among the shared aspects, high concentrations of voltage-dependent sodium channels at the nodes of Ranvier are linked to the cytoskeleton by ankyrin G, while the presence of Caspr (contactin-associated protein)/paranodin (a glycoprotein of neuronal paranodal membranes), the potassium channels Kv1.1 and Kv1.2, the associated β2 subunit, and Caspr2, characterize both the myelin sheaths. Among the differences, there are the cellular origins, the anatomic details and the molecular constituents. 

It has been demonstrated that biosynthesis, storage, and cellular trafficking of specific lipids involved in the formation of the myelin sheath are fundamental for its assembly and maintenance. The CNS myelin has a high lipid content (about 70%) made of galactosphingolipids, sphingomyelin and cholesterol. On the other hand, only a few proteins, specific to the CNS, compose the myelin sheath, but with high density, and among these are the proteolipid protein (PLP), myelin-associated glycoprotein (MAG) and MBP [11,12]. PLP is the most abundant transmembrane protein and a suggested explanation for the tight apposition of membrane sheaths, due to the hydrophilic extracellular domains. MBP is an unstructured polypeptide chain which binds to the membrane where it is necessary for the compaction of the two adjacent cytoplasmic membrane surfaces into the MDL. Another important protein is claudin-11, which is a tight junction protein and connects the outer leaflets of the myelin lamellae. CNS myelin does contain tight junctions, which enhance the insulative properties of myelin, but lacks the components of adherens and gap junctions [10].

During the synthesis of myelin, lipids preassemble, together with PLP, and their transport to the myelin sheath is mediated by vesicles and motor proteins [13]. 

The myelin membrane is defined as “compacted” when the extracellular and cytoplasmic leaflets of the adjacent myelin lamellae connect very tightly and most of the water is removed. Consequently, electron-dense lines can be seen by electron microscopy [10].

The PNS myelin is dependent on the Schwann cells which present a basal lamina and microvilli containing F-actin and potassium channels fundamental for axonal activity, and which are linked to the nodal axolemma. Contrary to the oligodendrocytes, the Schwann cells make a single internodal region of myelin [12]. In these cells, the endoplasmic reticulum and the Golgi apparatus are found in a perinuclear position, while most of the cytoplasm is external to the main part of the cell. Moreover, Schwann cells have myelin incisures, also known as Schmidt–Lanterman incisures, which are small pockets of residual cytoplasm histologically visible that are produced during the myelination process in the inner layer of the myelin sheath [14]. 

The “compact” myelin sheath forms the bulk of the myelin sheath, and the distance between two layers of cell membranes is lowered, while the lateral border of the sheath and the Schmidt–Lanterman incisures constitute the “non-compact” myelin sheath. Both the components contain a non-overlapping set of proteins. 

Compact myelin in the PNS is largely composed of lipids, mainly cholesterol and sphingolipids, including galactocerebroside and sulfatide. Interestingly, myelin protein 0 (P0) is the major protein component, together with Po-like proteins and peripheral myelin protein 22 (PMP22). P0 is a single membrane glycoprotein, and it is the most abundant protein in myelin. P0 has a structural role and it is required for the proper formation and maintenance of myelin, similarly to the transmembrane protein PMP22.

The Schwann cell basal lamina is composed of laminin 2 (comprised of α2/merosin, β1, and γ1 laminin chains), type IV collagens, entactin/nidogen, fibronectin, N-syndecan and glypican. Furthermore, it contains the integrin α6β4 and dystroglycan. The apical surface is highly enriched in MAG [14]. MAG is a type 1 transmembrane glycoprotein which functions in the glia-axon interaction and in the maintenance of myelinated axons. In the PNS, the non-compact myelin contains E-cadherin, MAG, and connexin32 (Cx32). Moreover, adherents and gap junctions are located at the plasma membrane. 

The neurotransmitter release from presynaptic neurons is one of the main events in synaptic transmission. The neurotransmitters are packed into synaptic vesicles which, upon activation of the neuron, move towards the synaptic cleft and release their content, which can bind specific receptors on the plasma membrane of postsynaptic neurons. The connections between the axons and dendrites of the adjacent neuron are named the synapses. The transmission of the information is based on the release of specific molecules through different systems [15]. This can be either by spontaneous vesicle fusion, which is independent of presynaptic action potentials, or by evoking the neurotransmitter release [16]. Molecular machinery triggering spontaneous vesicle fusion may differ from that underlying the evoked release, and may be one of the sources of heterogeneity in release mechanisms.

The myelin sheath is fundamental for the transmission of signals and the proper functionality of the nervous system, and therefore its alteration determines diseases, named demyelinating and dysmyelinating [17]. In the first case, the neurological disorders are divided into primary demyelination, where myelin and supporting cells are damaged or degraded, and secondary demyelination, where axonal damage determining the interruption of the axon–glial interactions necessary for the maintenance of the myelin sheaths, occurs. If the onset of the pathology is during development, the myelin is not formed and metabolic disturbances affect myelin synthesis or degradation (dysmyelinating disease). For instance, specific mutations in the Po gene cause CMT (Charcot–Marie–Tooth) type 1B, due to the alterations in the amount of Po protein. An extra copy of the PMP22 gene has been associated with CMT1A, presenting an increase in the amount of PMP22 in compact myelin. Other diseases due to an alteration of this gene or CX32 are hereditary neuropathy with liability to pressure palsies (HNPP), X-linked Charcot–Marie–Tooth disease (CMTX) [14] or multiple sclerosis, which is a CNS demyelinating disorder [12,18]. Besides the above-mentioned neurological pathologies, other diseases involving myelinated axons may occur, such as acquired inflammatory and infectious diseases of myelin, acquired toxic-metabolic diseases, nutritional diseases, hereditary metabolic diseases, and diseases of myelin due to physical agents [19].

## 2. SNAREs

The synthesis of the myelin sheath and the assembly of myelin proteins are regulated by neuronal signals. Interestingly, some soluble N-ethylmaleimide-sensitive factor attachment protein receptors (SNAREs) are an important component of the fusion machinery, and are involved in delivering molecules to the myelin sheath. SNAREs are involved in the regulation of vesicle fusion; they are normally membrane proteins and are involved in the docking and fusion between two vesicles or a vesicle and a specific compartment. SNARE proteins are divided into v-SNAREs (vesicle-SNARE) and t- SNAREs (target-SNARE), which locate on the two different membranes. SNAREs can be grouped also into R-, Qa-, Qb-, Qb-c-, or Qc- SNAREs, depending on the aminoacidic residue that is exposed in the formation of the zero ionic layer in the assembled core SNARE complex, which is formed by the assembly of three or four SNAREs in one unique complex when the fusion process is complete. The proteins which form the SNARE complex are located at different compartments, with two or three Q-SNAREs at one compartment and one R-SNARE on another compartment. For quaternary complexes, a Qa-, a Qb-, a Qc- and an R- SNARE are required. Ternary complexes are made only of a Qb-c, a Qa-, and an R- SNARE. Several combinations of R- and Q-SNAREs take part in specific functions [20]. For instance, vamp-2 (vesicle-associated membrane protein 2), together with syntaxin 1 and SNAP-25 constitute a ternary complex in neurons for synaptic vesicle fusion, syntaxin 7, syntaxin 8, vti1b and vamp8 are components of a quaternary complex responsible for the fusion of early endosomes with late endosomes, and syntaxin 7, syntaxin 8, vti1b and vamp7 are the SNAREs responsible for the fusion between late endosomes and lysosomes [21].

For the formation of the myelin sheath, some important events occur, such as the MBP transport and the exocytosis of PLP. The role of several SNARE proteins in this process has been demonstrated. In the work of Feldmann and colleagues [22], it has been proved that the transport of PLP is regulated by two different R-SNARE vamps (vesicle-associated membrane proteins), vamp3 (or cellubrevin) and vamp7 (also named TI-VAMP). Both of these colocalized with PLP in different compartments, recycling endosomes (RE) in the case of vamp3 and late endosomes and lysosomes in the case of vamp7. In particular, immune electron microscopy on primary cultures proved the colocalization of vamp3 and PLP on RE on the ultrastructural level [22]. Putative components of the complex including this SNARE protein were syntaxin 4 and snap23 (synaptosomal-associated protein 23). A role for vamp3 in PLP transport in the secretory pathway was demonstrated, as it mediated the fusion of RE-derived vesicles with the plasma membrane of oligodendrocytes [22]. Interestingly, vamp3 mRNA expression and protein levels were upregulated during the differentiation process. Nevertheless, the study of vamp3-deficient mice did not show any myelination defects [22].

Similarly, vamp7 localization and function also determined an enrichment of PLP in the myelin membrane during myelination, as it controls the exocytosis of PLP from late endosomes/lysosomes in a transcytosis pathway [22]. Syntaxin 3 and snap23 have been identified as prime acceptors of vamp7. Interestingly, the functional inactivation of vamp7 decreased PLP surface transport [23]. Therefore, the role of vamp7 is linked to the exocytosis of lysosome-related organelles and the transport of cargo to the myelin membrane in order to promote myelin biogenesis [23]. PLP was enriched in the myelin membrane during myelination. In order to study the role of vamp7 in vivo, the authors used mice deficient in a subunit of the adaptor protein 3 (AP-3) which determined a lack of functional AP-3 and the mislocalization of vamp7 to early endosomes, therefore altering secretion [23]. Consequently, immunohistochemical staining of PLP showed a lower signal in the striatum and the hippocampus, where the signal of myelinated fine fibers was extremely low [23]. 

Interestingly, silencing of vamp3 or vamp7 in primary oligodendrocytes determined a lower amount of PLP on the plasma membrane, and they were found to be able to have a synergistic effect [23]. 

Moreover, another Q-SNARE, named snap29, has been associated in complex with syntaxin 6 and vamp4 with the surface transport of PLP in oligodendrocytes, through the interaction with rab3a (ras (rat sarcoma)-associated binding protein 3) [24]. Snap29 is abundant in oligodendrocytes during myelination and in the noncompact myelin of the peripheral nervous system. Moreover, the two proteins overlapped in glia and neurons, and were recruited together from the periphery of the cell where snap29 is normally located toward the perinuclear membrane localization when active rab3a was present; therefore, snap29 was considered to be an effector of the GTPase protein (guanine nucleotide-binding protein, an enzyme that catalyzes the hydrolysis of guanosine triphosphate (GTP) to guanosine diphosphatase (GDP)) [24]. Both rab3a and snap29 enhanced the trafficking of PLP to the plasma membrane. Snap29 and rab3a expression were linked to the remyelination process, as it has been proved that, in the cases of pathologies such as sciatic nerve crush and Charcot– Marie–Tooth 1A (CMT1A), the abundance of snap29 and rab3a followed the expected reduced and restored abundance during demyelination and remyelination that would occur in the proteins involved in myelin formation [24]. Besides rab3a, snap29 also interacts with rab24 and sept4 (septin 4), which is a member of a family of GTP-binding proteins involved in regulating the cytoskeletal organization localized in the mitochondria, and has a role in apoptosis and cancer [24].

It has been demonstrated that syntaxin 4 is fundamental for the transcriptional expression of MBP. This protein localizes at the cytoplasmic surface of the myelin membranes where the SNARE protein is located, while syntaxin 3 is localized mainly in the cell body. MBP is a basic and membrane-associated adhesive protein and it is transported to the myelin sheath in a syntaxin 4-dependent mechanism in its mRNA form, which should avoid the premature adhesion of membranes. Subsequently, MBP mRNA is assembled in nonmembranous granules. Syntaxin 4-mediated autocrine signaling was necessary for initiating MBP mRNA transcription. Syntaxin 4 is upregulated during the differentiation of oligodendrocytes, while its downregulation determined the block of MBP mRNA transcription which was rescued by a conditioned medium from developing oligodendrocytes. Additionally, in this case, syntaxin 4 formed a complex with vamp3. Interestingly, the downregulation of vamp3 determined a reduction in MBP expression levels and a slight increase in the amount of syntaxin 4. The downregulation of syntaxin 3 did not affect the expression of MBP, but it mislocalized PLP to the cell body [25]. 

Interestingly, in a snap25 knock-out mouse, where snap25 is necessary for regulated synaptic vesicle release and has three isoforms (snap23, snap25a and snap25b), the inhibition of regulated vesicular trafficking in the layer VI cortical projection neurons decreased the MBP and the amount of myelinated projections at postnatal day 14 (P14), but it did not affect the initial timing of the onset of myelination in the brain (P7/P8). Oligodendrocyte maturation was therefore affected [26].

Besides PLP and MBP, other proteins and lipids are necessary for myelin sheath formation. They are synthetized, directed and trafficked toward myelin, and membrane expansion within myelin sheaths can occur through several mechanisms, such as vesicular trafficking to the cell surface, non-vesicular lipid transport, or membrane incorporation of lipoproteins. Lam and colleagues have demonstrated that oligodendrocytes may add new membrane through the action of vamp2 and vamp3 SNAREs by initiating wrapping and power sheath elongation, and that these SNAREs were required for node of Ranvier assembly. Moreover, membrane expansion has been associated with axon–myelin adhesion protein transport by vamp2 and vamp3 to the inner tongue and paranodes to assemble the nodes of Ranvier. Among the proteins that might be affected by vamp2 and vamp3 were contactin-1 (Cntn1), neurofascin (Nfasc), and the myelin-associated glycoprotein (MAG). The first two were necessary for the establishment of axon–myelin junctions at paranodes, whereas MAG maintains axon–myelin interactions at the internodes. Furthermore, it has been demonstrated that genetic inactivation of vamp2 and vamp3 in myelinating oligodendrocytes was responsible for severe hypomyelination and premature death without oligodendrocyte loss [27].

**Figure 1:**
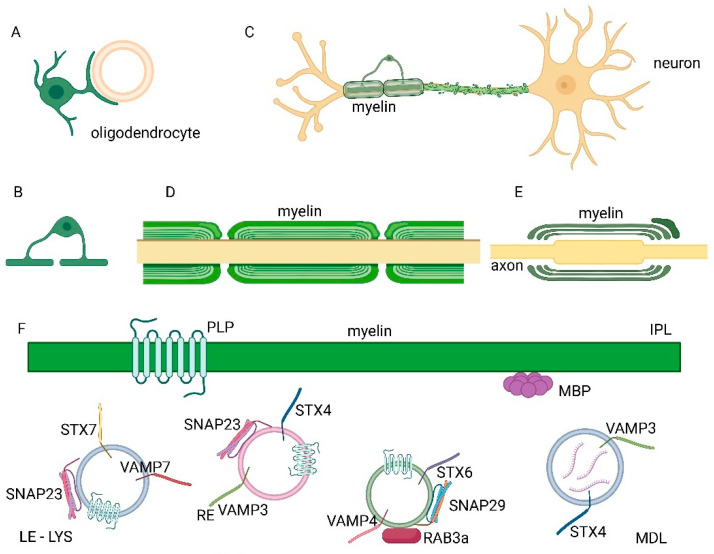
Myelination and protein transport to myelin, mediated by soluble N-ethylmaleimide-sensitive factor attachment protein receptors (SNAREs) in the central nervous system (CNS). (**A**) An oligodendrocyte during myelination. (**B**) Each oligodendrocyte can form multiple sheaths around several axons. (**C**) Neuron showing a partially myelinated axon by an oligodendrocyte and the demyelination process. (**D**) Longitudinal view of the axon and the myelin sheaths. (**E**) Longitudinal section of the axon and non-compacted myelin wrapping. (**F**) Vesicle transport of protein components of the myelin sheath, such as proteolipid protein (PLP) and myelin basic protein (MBP), is regulated by SNARE proteins such as vamp7 (vesicle-associated membrane proteins), stx7 (syntaxin 7) and snap23 (synaptosomal-associated protein 23) transport of PLP in late endosomes (LE)- lysosomes (LYS), stx4, vamp3 and snap23 in recycling vesicles (RE) and by the SNARE complex composed of stx6, vamp4 and snap29 and the rab3a (ras (rat sarcoma)-associated binding protein 3a) GTPase (guanine nucleotide-binding protein). Major dense line (MDL) and intraperiod line (IPL) are indicated in F. Vesicles and relative SNARE proteins, which form a complex, are shown close to the plasma membrane where the fusion of the membranes occurs. Membrane fusion is regulated by rab proteins which can associate with the vesicles.

Taking all this into consideration, the expression of some SNAREs, such as syntaxin 3, syntaxin 4, and vamp3, is linked to that of myelin markers and myelination. While their abundance increases, that of snap29 decreases. Other affected proteins are vamp4, syntaxin 2, syntaxin 8, and snap23. Furthermore, the maturation and the accomplishment of myelinated axons is associated with syntaxin 3, syntaxin 4, snap23 and vamp3. Moreover, syntaxin 3, syntaxin 4, and vamp7/tetanustoxin-insensitive vamp accumulated in myelin during development, suggesting a role in myelin membrane fusion, while the increase in expression of vamp2 with maturation is most likely due to its abundance in the synapses [23] (Figure 1).

## 3. RABs

**Figure 2:**
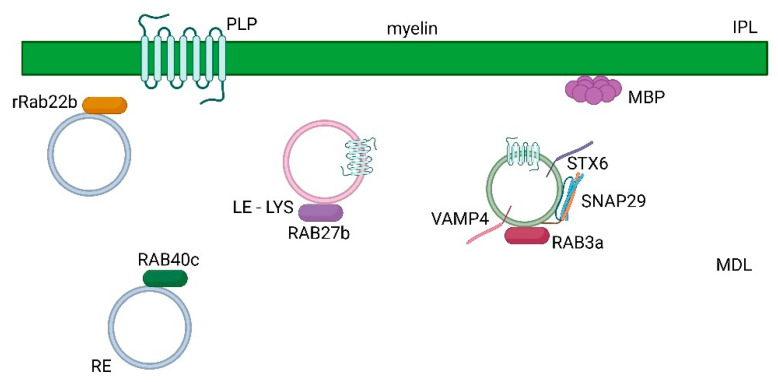
Formation of myelin sheath mediated by rab proteins (ras (rat sarcoma)-associated binding protein) in the central nervous system (CNS). Vesicle transport of protein components of the myelin sheath, such as proteolipid protein (PLP) and myelin basic protein (MBP), is regulated by rab proteins such as rab27b transport of PLP in late endosomes (LE)- lysosomes (LYS), rab40c in recycling compartments (RE), rrab22b and by rab3a GTPase and the SNARE complex composed of stx6, vamp4 and snap29. Major dense line (MDL) and intraperiod line (IPL) are indicated.

The synthesis of the myelin sheath and the assembly of myelin proteins are regulated by rab proteins through the modulation of the intracellular trafficking and fusion of vesicles. Several rabs have been discovered to affect important processes for myelin growth (Figure 2). Rabs are small GTPases belonging to the Ras superfamily, and are involved in the regulation of the intracellular trafficking and the fusion of vesicles between different compartments [28]. Rab GTPases are molecular switches, cycling between an active GTP-, membrane-bound form, and an inactive, cytosolic, GDP-bound form. Rabs exert their functions by interacting with specific effectors [29].

In oligodendrocytes the following rab proteins are expressed: rab1, rab1b and rab2 in the early secretory pathway, rab8 and rab12 in the late secretory pathway, and rab3a and rab3b in the regulated exocytic pathway. Furthermore, rrab22b, a rat rab protein cloned from an oligodendrocyte cDNA library in 2001 by Rodriguez-Gabin and colleagues [30], regulates the transport from the trans-Golgi network to endosomes, and rab5a, rab5b, rab5c, rab7 and rab9 are involved in the endocytic process. Rab14, rab18, rab23, rab24, rab26, rab40c and rab28 are also expressed in oligodendrocytes [30,31,32,33,34,35,36,37,38,39].

Interestingly, SNAP-29, a SNARE abundant in oligodendrocytes during myelination and in Schwann cells, interacts with different rab proteins, such as rab3a and rab24, with its N-terminal domain in a GTP-dependent or GTP-independent manner. As previously mentioned, rab3a and snap29 colocalized both at the synapses and in the myelinating glia, and their expression enhanced the surface-directed trafficking of the myelin proteolipid protein [24]. 

Rab40c is a small GTPase that localized in the perinuclear recycling compartment (PRC), and it has been linked to endocytic events such as receptor recycling and myelin formation, as both rab40c mRNA and protein increase as oligodendrocytes differentiate. Interestingly, rab40c mRNA expression was identified in primary cultures of oligodendrocytes, oligodendrocyte progenitors, microglia, and astrocytes [39]. 

Other rabs that are involved in myelin biogenesis and maintenance and oligodendrocyte differentiation are rab27b, rab35 and rrab22b. Rab35 and its effector, ACAP2 (ArfGAP (GTPase-activating protein) with coiled-coil, ankyrin repeat and PH domains 2), are downregulated during the differentiation of oligodendrocytes, affect the arf6 (ADP (adenosine diphosphate)-ribosylation factor 6) activity in the trafficking of biological membranes and transmembrane protein cargo, and promote myelination. This event is mediated also by the role of cytohesin-2 [40]. The transport of certain proteins from the trans-Golgi to myelin was mediated by small tubule vesicular organelles containing the rat Rab protein rrab22b. These vesicles have been associated with areas close to the axons, where the active formation of myelin occurred [30].

One of the rab proteins which has a role in PLP transport is rab27b. Rab27b colocalized with PLP in late endosomes/lysosomes of mature oligodendrocytes, and it regulated its surface transport and exocytosis. In particular, silencing of rab27b determined the reduction of lysosomal exocytosis and therefore regulated the surface expression of the myelin protein in oligodendrocytes and its release from myelin-like membrane (MLM) formation in oligodendrocytes neuron co-cultures [41]. 

**Figure 3:**
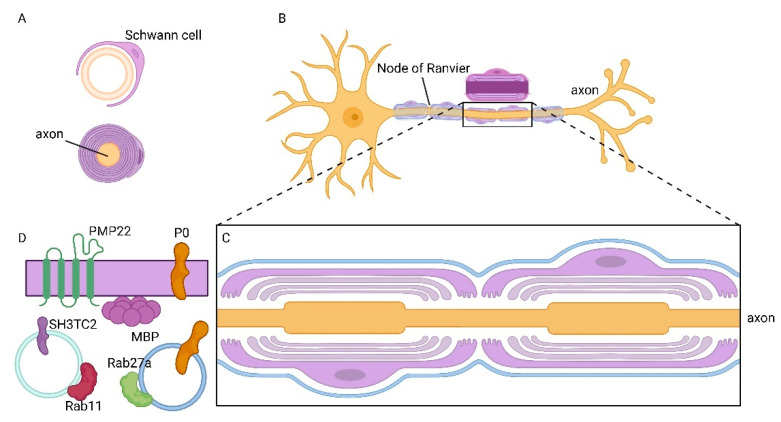
Formation of myelin sheath mediated by rabs (ras (rat sarcoma)-associated binding protein) in the peripheral nervous system (PNS). (**A**) The plasma membrane of a Schwann cell forms the myelin sheath. (**B**) PNS neuron and Schwann cells enveloping the axons and making the myelin sheath. (**C**) Enlargement of an axon by two Schwann cells forming a non-compacted myelin sheath. (**D**) Transport of proteins which are components of the myelin sheath, mediated by vesicles and Rab proteins. Rab11 regulates the transport of SH3TC2, whereas rab27a is responsible for the trafficking of myelin protein 0 (P0).

Interestingly, it has been demonstrated that rab proteins can regulate the demyelination of Schwann cells (Figure 3). Indeed, altered rab11-dependent endocytotic trafficking of the laminin receptor SH3TC2 downregulated myelination, determined denervation, and affected its maintenance. The myelination process is dependent on the expression of specific isoforms of the integrin receptors. The most important ones are α6β1 and α6β4, which are related to the early step of the myelination process and the structural stability of the mature myelin sheath. The endocytic recycling of the α6β4 complex regulates its surface expression [42].

Similarly, downregulation of rab27a, which regulates the trafficking of the secretory lysosomes to the plasma membrane, inhibited the lysosomal exocytosis in Schwann cells and reduced the remyelination of the regenerated sciatic nerve by modulating the trafficking of the myelin protein P0 in late endosomes and lysosomes [43]. 

Rab35 modulates endosomal trafficking, the secretion of extracellular vesicles, actin dynamics, cell migration and cell signaling. Moreover, rab35 controls myelin sheath synthesis by forming a complex with the myotubularin-related phosphatydilinositol (PI) 3-phosphatase MTMR13 and MTMR2, whose mutated forms have been associated with CMT4 (Charcot–Marie–Tooth type 4) B2 and B1 diseases in humans. In particular, rab35-GTP recruited MTMR13-based lipid phosphatase complexes. Rab35 was able to down-regulate the lipid-mediated mTORC1 (mammalian target of rapamycin complex 1) activation, which regulates cell growth and metabolism by modulating growth factors and amino acids, and alter the myelin biogenesis. Disruption of rab35 activity determined the hyperactivation of mTORC1 signaling, due to elevated levels of PI 3-phosphates and hypermyelination and loss of rab35 in the Schwann cells. Therefore, the synthesis of the myelin sheath was altered [44]. 

## 4. Conclusions

The myelin sheath is an insulating layer composed of lipids and proteins, and it constitutes a protective cover for the nerves in the CNS and the PNS. Myelin can be damaged in different ways, and the nerves’ ability to send and receive electrical impulses can be slowed or stopped. The demyelinating process of the brain can be a consequence of brain injury, aging or stroke. It occurs in many neurodegenerative diseases, and the death of oligodendrocytes is one of the reasons for white matter damage. In healthy brains, oligodendrocytes precursors cells (OPCs) differentiate into mature oligodendrocytes, and are responsible for remyelination. The failure of this process is the main problem for brain repair [45].

In the PNS, the perturbation of myelin structure and function causes axonal demyelination or dysmyelination. Myelin is fundamental for axonal integrity and fast axon potential propagation, and damages to the myelin have been associated with Charcot–Marie–Tooth disease, where mutations to the PMP22, P0, and gap junction beta 1 (coding for connexin 32) are responsible for CMT1A, CMT1B and X-linked CMT1 disease, respectively [46].

The regulation of the transport of specific molecules to the myelin sheath for proper biogenesis, maturation and functionality is fundamental in order to avoid demyelination or dysmyelinating processes. The role of Rab and SNARE proteins in neurological diseases has already been proved [47,48] and the interplay of these two classes of proteins in membrane fusion can be important for elucidating novel mechanisms in the regulation of molecule trafficking [49]. Therefore, the role of SNAREs and rabs in processes involving the biogenesis, maturation and functionality of the myelin sheath, in events that negatively affect the nerve conduction such as demyelination and dysmyelination, and in positive biological processes, such as remyelination, is fundamental for future therapeutic approaches and for counteracting the progression of related diseases.
